# Role of NRF2 in Lung Cancer

**DOI:** 10.3390/cells10081879

**Published:** 2021-07-24

**Authors:** Miriam Sánchez-Ortega, Ana Clara Carrera, Antonio Garrido

**Affiliations:** Department of Immunology and Oncology, Centro Nacional de Biotecnología, Consejo Superior de Investigaciones Científicas (CSIC), Universidad Autónoma de Madrid, Cantoblanco, E-28049 Madrid, Spain; msanchez@cnb.csic.es (M.S.-O.); acarrera@cnb.csic.es (A.C.C.)

**Keywords:** NRF2 activation, NSCLC, therapeutic strategies

## Abstract

The gene expression program induced by NRF2 transcription factor plays a critical role in cell defense responses against a broad variety of cellular stresses, most importantly oxidative stress. NRF2 stability is fine-tuned regulated by KEAP1, which drives its degradation in the absence of oxidative stress. In the context of cancer, NRF2 cytoprotective functions were initially linked to anti-oncogenic properties. However, in the last few decades, growing evidence indicates that NRF2 acts as a tumor driver, inducing metastasis and resistance to chemotherapy. Constitutive activation of NRF2 has been found to be frequent in several tumors, including some lung cancer sub-types and it has been associated to the maintenance of a malignant cell phenotype. This apparently contradictory effect of the NRF2/KEAP1 signaling pathway in cancer (cell protection against cancer versus pro-tumoral properties) has generated a great controversy about its functions in this disease. In this review, we will describe the molecular mechanism regulating this signaling pathway in physiological conditions and summarize the most important findings related to the role of NRF2/KEAP1 in lung cancer. The focus will be placed on NRF2 activation mechanisms, the implication of those in lung cancer progression and current therapeutic strategies directed at blocking NRF2 action.

## 1. Introduction

**Figure 1:**
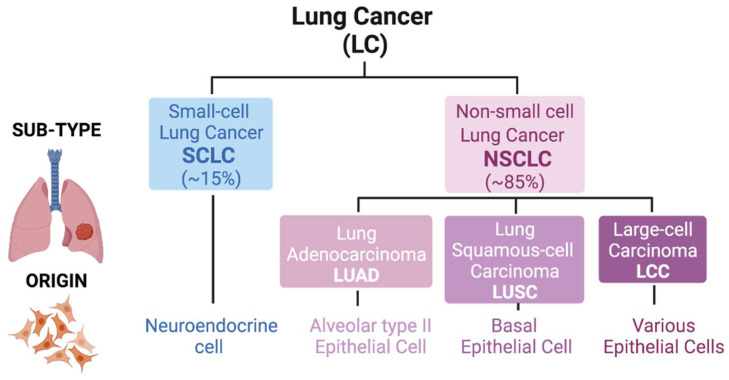
Classification of lung cancer. Lung tumors are divided into two main groups: Small-cell Lung Cancer (SCLC; ≈15% of cases) and Non-small cell Lung Cancer (NSCLC; ≈85% of cases). An SCLC tumor derives from neuroendocrine cell linage. However, in NSCLC, the tumor origin cell is different, being classified into three different sub-types: Adenocarcinoma (LUAD; alveolar type II epithelial cell), Squamous-cell Carcinoma (LUSC; basal epithelial cell) and Large-cell Carcinoma (LCC; various epithelial cells).

Lung cancer, one of the most commonly diagnosed malignancies, has some of the lowest 5-year survival rates and is responsible for around 20% of all cancer-related deaths worldwide [1,2]. Histologically, this malignancy is classified into two groups: small-cell lung cancer (SCLC) and non-small-cell lung cancer (NSCLC) (the latter being the most frequent, around 85%). In small-cell lung cancer, the tumor derives from cells of the neuroendocrine lineage upon loss of *RB* and *TP53*, whereas in non-small sub-types, the tumor originates from lung epithelia after distinct genetic events. NSCLC is further divided into three sub-types based mainly on the morphology of the transformed cells: adenocarcinoma (LUAD), squamous-cell carcinoma (LUSC) and large-cell carcinoma (LCC) [3]. The observation that LUAD typically arises in the distal lung, whereas LUSC arises centrally, probably reflects different cells-of-origin for these two lung cancer sub-types [4]. It is widely accepted that LUAD develops from alveolar type II (AT2) epithelial cells or cells within bronchioalveolar duct junctions, whereas LUSC develops from basal epithelial cells in airways [5]. LUSC differentiates into a stratified squamous epithelium that is not found in non-keratinizing epithelia. The third NSCLC sub-type, LCC, also originates from lung epithelial cells and represents a heterogeneous group of malignant neoplasms that lack the cytological and architectural characteristics of small cell and glandular carcinoma (LUAD) or squamous (LUSC) sub-types. All forms of lung cancer have poor prognosis, particularly SCLC and LUSC, which are typically observed in tobacco smokers [6]. LCC has a relatively better prognosis (depends on sub-type). While LUAD already has several available targeted therapies, SCLC has some therapies currently under study [7]. In the case of LUSC, there is an urgent need for development of targeted therapies (Figure 1).

During the past half-century, different bioinformatic and next-generation sequencing analyses of data from large patient cohorts have permitted the identification of key genes involved in the generation and progression of many tumor types [8,9]. In this context, the Cancer Genome Atlas (TCGA) consortium and other projects have found that some genes related to antioxidant regulatory mechanisms, including the genes encoding for NRF2, *NFE2L2* (nuclear factor erythroid 2-related factor 2) and its negative regulator, *KEAP1* (Kelch-like ECH-associated Protein 1), are altered in several lung cancers [10,11,12]. In particular, gain-of-function (GOF) mutations of *NFE2L2* or loss-of-function (LOF) mutations of *KEAP1* are frequent in NSCLC tumors [13]. Indeed, *KEAP1* mutations are frequently found in LUAD (17%) and LUSC (12%), whereas *NFE2L2* mutations are more common in LUSC (15%) compared to LUAD (3%) [10]. Moreover, 26% of NSCLC tumors present high expression of nuclear NRF2 [14], with a higher incidence in the LUSC sub-type [10]. In LCC, this mutational phenomenon in *NFE2L2/KEAP1* is less common [15,16,17].

Based on initial studies, NRF2 was first considered a tumor suppressor gene, since a number of studies in other cancer types (i.e., colon, melanoma) and in mice showed that *NFE2L2* deficiency increases the susceptibility to cancer [18,19,20]. More recently, the linkage of GOF alterations in *NFE2L2* with cancer progression and an inefficient response to classical cancer treatments (radiotherapy and chemotherapy) in tumors with active NRF2 [21,22,23] support that NRF2 might act as a tumor driver in several cancer sub-types (particularly in LUAD and LUSC). This dual role of NRF2 as tumor suppressor or driver, depending on the tumor type or stage, has given rise to a substantial debate about its role in cancer [13].

In this review, we will describe how this signaling pathway works in physiological conditions and summarize the most important findings related to the role of NRF2/KEAP1 in LUAD and LUSC, focusing on their activation mechanisms and their implications in the progression of cancer. In addition, current therapeutic strategies developed to target NRF2 in lung cancer will be considered.

## 2. NRF2/KEAP1 System in Physiological Conditions

Aerobic organisms require oxygen for survival; nonetheless, these organisms generate oxygen byproducts named reactive oxygen species (ROS) [24]. These oxygen byproducts together with reactive nitrogen species (RNS) regulate a variety of cellular responses [25]. However, these oxidized species have to be tightly regulated as an excess of ROS/RNS introduces oxidative damage to proteins, lipids and DNA and in turn to genetic and/or epigenetic alterations, which might lead to cell death [24]. As an adaptation to this oxidant environment, cells have developed a variety of antioxidant systems to keep cellular ROS levels under certain limits. NRF2 acts as a master regulator of these antioxidant mechanisms since it is responsible for the activation of several transcriptional programs in response to cellular oxidative stress [26].

**Figure 2:**
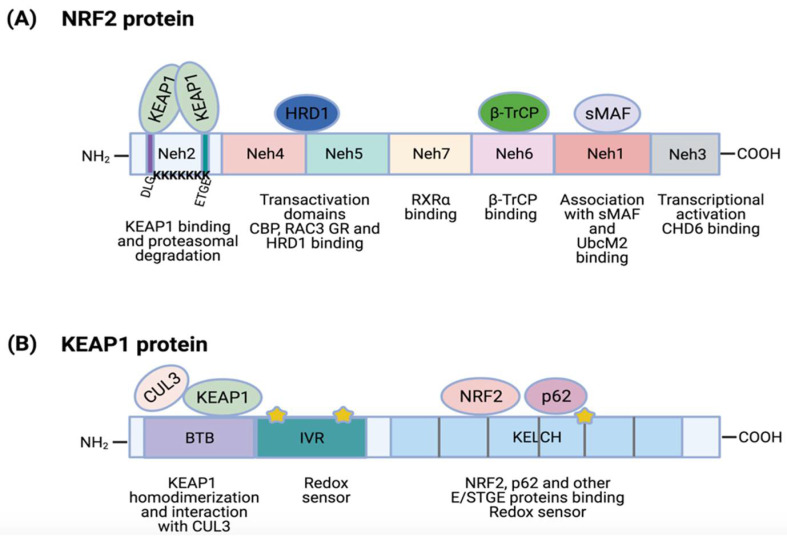
NRF2 and KEAP1 protein structures. (**A**) NRF2 contains 7 highly conserved domains called Neh domains. Neh1 is required for complex formation with transcription factor sMAF, DNA binding and for binding to UbcM2. Neh2 contains ETGE and DLG sequences that are required for KEAP1 binding and 7 ubiquitin-lysine residues for targeting NRF2 for proteasomal degradation. Neh3 is needed for transcriptional activation (CHD6 binding). Neh4 and Neh5 are transactivation domains that bind activators (CBP, RAC3) or repressors (GR, HRD1). Neh6 regulates NRF2 stability by binding to β-TrCP. Finally, Neh7 can interact with RXRα, an NRF2 repressor; (**B**) KEAP1 protein contains 5 conserved regions: N-terminal region, BTB, IVR region, DGR domain and C-terminal region; DGR and C-terminal regions form a Kelch motif. The BTB domain facilitates KEAP1 homodimerization and CUL3 binding. The IVR region possesses a cysteine-rich domain that acts as a direct redox sensor. DGR/Kelch regions contains 6 repeats of a Kelch motif that mediate their interaction with NRF2 and other proteins with E/STGE conserved motifs, such as p62. This region also contains additional cysteine residues for stress sensing. Stars represent several cysteine residues located in IVR and Kelch domains.

NRF2, encoded by *NFE2L2*, is a transcription factor that belongs to a protein family characterized by the presence of a cap ‘n’ collar (CNC) homology domain [27]. NRF2 is a 605 amino acid protein with a molecular weight of 68 kDa that contains seven highly conserved NRF2-ECH (erythroid-derived cap ´n´ collar homology, Neh) domains [28] (Figure 2A).

The Neh1 is a basic region leucine-zipper motif required for DNA binding at antioxidant response elements (ARE; also named EpRE, electrophile response elements) of gene promoters and for NRF2 dimerization with other transcription factors [13,28]. The Neh1 region also regulates NRF2 stability by its interaction with the ubiquitin-conjugating enzyme, UbcM2 [29]. The Neh2 domain is located at the N-terminal region and it contains the ETGE (from aspartic acid, threonine, etc.) and DLG amino acid motifs, which are essential for the binding to KEAP1 [30]. In addition, the Neh2 domain contains seven ubiquitin-accepting lysine residues that mediate NRF2 proteasomal degradation [31]. In the C-terminal region is the Neh3 domain, needed for transcriptional activation. In fact, chromo-ATPase/helicase DNA binding protein family member (CHD6) has been identified to directly associate with the Neh3 domain via a VFLVPK motif [32]. Both Neh4 and Neh5 domains are two independent transactivation domains [33]. Co-activators such as CREB-binding protein (CBP) or receptor-associated co-activator 3 (RAC3) are able to bind to Neh5 to promote an increase in NRF2 expression [33,34]. Nevertheless, Neh4 and Neh5 can also bind other transcriptional regulators, such as glucocorticoid receptor (GR). Alam and colleagues showed that binding of GR to Neh4 and/or Neh5 domains can displace Histone acetyltransferase CBP and thus suppress H3K27 acetylation in the promoter of *NFE2L2* target genes to produce a reduction of *NFE2L2* transcriptional program [35]. The Neh6 domain is a serine rich region that is involved in the regulation of NRF2 stability [13]. It contains two amino acid motifs (DSGIS and DSAPGS), which are binding sites for the β-transducin repeat-containing protein (β-TrCP) that promote NRF2 poly-ubiquitination [36,37]. Finally, the Neh7 domain interacts with an NRF2 repressor, the retinoic X receptor α (RXRα), for the inhibition of *NFE2L2* target gene transcription [38].

As mentioned earlier, the activity of NRF2 is negatively regulated by KEAP1, a substrate adapter protein for the E3 ubiquitin ligase complex CUL3/RBX1 (complex of human cullin-3 with human RING box protein 1) [24,39,40,41]. This regulatory role of KEAP1 was confirmed in animal models as *Keap1* knockout mice showed constitutive activation of Nrf2 and sustained expression of Nrf2 target genes [42]. KEAP1 protein is widely expressed in different cell types and tissues and localizes in the cytoplasm perinuclear region, endoplasmic reticulum and to a lesser extent in the nucleus [43].

KEAP1 belongs to the BTB-Kelch protein family, which contains two canonical domains in common: a BTB domain (Broad-Complex, Tramtrack and Bric a brac) and a Kelch domain (domain present in Kelch proteins). The KEAP1 primary structure comprises 5 regions (depicted in Figure 2B): an N-terminal region (NTR), the BTB domain, an intervening region (IVR), a double-glycine repeat (DGR) domain and the C-terminal region (CTR); DGR and C-terminal domains form a Kelch domain (321–624) [44]. The BTB domain is needed for KEAP1 homodimerization and for the interaction with CUL3 [44,45]. The IVR region is a cysteine (Cys)-rich region containing direct redox sensors. The positively charged environment of basic amino acids K131, R135, K150 and H154 near to the Cys-rich region of the IVR region is responsible for the high reactivity of these Cys [46]. Next to it, there is the DGR/Kelch domain containing up to six repeats of the Kelch motif forming a six-bladed β-propeller structure that mediates its interaction with other proteins. The DGR/Kelch domain binds to the ETGE (or STGE) and DLG amino acid motifs of different partners including the NRF2 Neh2 domain [41,47,48] p62, B-cell lymphoma extra-large (Bcl-xL), dipeptidyl Peptidase 3 (DPP3), splicing factor, arginine/serine-rich 10 (SFRS10), D-site of albumin promoter binding protein (DBP), etc. [44]. The Kelch domain also contains several Cys residues involved in ROS stress sensing [13]. Indeed, human KEAP1 contains up to 27 Cys, around twice more than an average human protein [44]. Under oxidative stress, these Cys are oxidized, resulting in an ideal stress sensor for oxidants and/or electrophiles [49].

### 2.1. Canonical Pathway of NRF2/KEAP1

**Figure 3:**
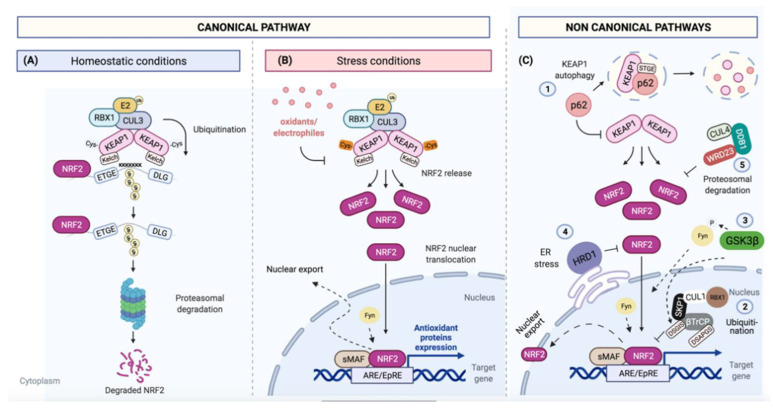
Canonical and non-canonical NRF2 pathways. (**A**) Under basal conditions, NRF2 ETGE/DLG motifs bind to KEAP1 Kelch domains. Binding to KEAP1 brings the CUL3/RBX1 E3 ubiquitin ligase into the complex and targets NRF2 for poly-ubiquitination and degradation by 26S proteasome; (**B**) Several oxidative and electrophilic stressors can modify critical KEAP1 cysteine residues, disrupting the KEAP1-NRF2 complex. As a consequence, NRF2 protein levels increase, causing its translocation into the nucleus where it forms a heterodimer with sMAF transcriptional factors to act on ARE/EpRE enhancer sequences for the control of its transcriptional program. Afterwards, NRF2 is exported to cytoplasm upon phosphorylation by different Src family kinases, such as Fyn; (**C**) NRF2 can be also regulated by KEAP1-independent mechanisms: (1) by p62/SQSTM1, which promotes KEAP1 autophagic degradation via its STGE binding motif; (2) by β-TrCP that can form a complex with CUL1/SKP1, promoting NRF2 ubiquitination and degradation; (3) by GSK-3β, which can phosphorylate β-TrCP, increasing NRF2 ubiquitination; (4) by HRD1, which can interact under reticulum stress conditions with Neh4 and 5 domains and trigger NRF2. Recently, (5) CUL4/DDB1/WDR23 was discovered as another E3 ubiquitin ligase to regulate NRF2, but its mechanism is still unclear.

NRF2 protein has a short half-life [50]. In fact, under normal (unstressed) conditions, protein levels of NRF2 are usually low as KEAP1 binds to NRF2 for its CUL3/RBX1 E3 ubiquitin-dependent degradation [51]. This KEAP1/NRF2 interaction is explained in the ´hinge and latch´ model: each Kelch domain of KEAP1 binds to the NRF2 protein by a strong-binding ETGE motif (hinge) and a weak-binding DLG motif (latch), the first binding affinity being around 100-fold higher than the second one [31,52]. In this binding, NRF2/KEAP1 complex adopts two different conformations: in the open conformation, newly synthesized NRF2 binds by ETGE motif to one KEAP1 molecule, but it is not until the binding of a second KEAP1 molecule by DLG motif that a closed conformation is acquired, predisposing NRF2 to its poly-ubiquitination and degradation by the 26S proteasome [53] (Figure 3A).

Several oxidative and electrophilic cellular stressors can directly modify critical KEAP1 Cys residues (Cys257, Cys273, Cys288, Cys297 and Cys151) by oxido-reduction and/or alkylation reactions [54,55]. Their redox modifications cause a conformational change that disrupts Kelch<>DLG binding, avoiding the NRF2 degradation by the proteasome. Electrophilic modifications of KEAP1 Cys are accompanied by KEAP1 degradation by autophagy mechanisms [50]. The binding between KEAP1 and CUL3 can be also disrupted by inhibition of CUL3 neddylation [56]. CUL3 acts as a scaffold protein that binds to BTB domain of KEAP1, allowing the formation of a complex with an E2 ubiquitin-conjugating enzyme [26]. Indeed, the complete inhibition of CUL3 neddylation leads to the cellular accumulation of NRF2 [56].

Under ROS stress conditions, KEAP1 Cys are oxidized and KEAP1 is released from NRF2 causing an increase in NRF2 levels and its translocation to the nucleus where it forms a heterodimer with sMAF transcriptional factors (v-Maf avian musculoaponeurotic fibrosarcoma oncogene homolog) or Jun proteins (c-Jun, Jun-B and Jun-D) [13,57]. NRF2 binds to ARE/EpRE sequences [28]. The ARE/EpRE are cis-acting DNA enhancer sequences with the consensus sequence: 5′-RTGABnnnGCR-3′ (“n”, any nucleotide) [58].

Among NRF2 target genes, there are drug metabolizing enzymes (phase I-III), redox response transcription factors, anti-apoptotic proteins, carbohydrate and lipid metabolizing enzymes, cell cycle regulators, proliferation regulators, proteostasis machineries (regulators of autophagy and proteasomal degradation), heme and iron metabolizing proteins and xenobiotic transporters [58,59]. Therefore, the NRF2 activation protects cells from a broad variety of cellular stresses, most importantly oxidative stress [50]. It is important to highlight that the promoter region of *NFE2L2* gene also contains an ARE sequence, providing the possibility of a positive feedback regulation, increasing the transcription of its target genes and promoting a fast response to any cellular stress [60].

After its transcriptional activation, NRF2 is exported from the nucleus to the cytoplasm; this transport is regulated by tyrosine phosphorylation on several residues. Members of Src subfamily A, like Fyn, are able to phosphorylate NRF2 in the nucleus for its nuclear export and degradation [61] (Figure 3B).

### 2.2. Non-Canonical Pathways

NRF2 can be also regulated by KEAP1-independent mechanisms. The ubiquitin binding autophagy receptor p62/sequestosome-1 (p62/SQSTM1) was identified as a regulator of ARE-element gene expression, independent of the cellular redox state [40]. p62/SQSTM1 binds to KEAP1 via its STGE motif and promotes its degradation, in turn increasing NRF2 protein levels [37]. Additionally, the E3 ubiquitin ligase complex formed by Cullin 1/S-phase kinase-associated protein 1/β-transducin repeat-containing protein (CUL1/SKP1/β-TrCP) can also regulate NRF2 levels [62]. β-TrCP serve as substrate recognition subunits for the SCF^β-TrCP^ (Skp1-Cullin1-F-Box protein) E3 ubiquitin ligases, resulting in NRF2 ubiquitination and degradation [36].

Another regulator of NRF2 is glycogen synthase kinase 3 beta (GSK-3β). This protein can phosphorylate β-TrCP, increasing the ability of β-TrCP to ubiquitinate NRF2 [36,50]. GSK-3β also regulates the phosphorylation and nuclear translocation of the tyrosine kinase Fyn, which in turn phosphorylates NRF2, promoting its return to the cytosol and degradation [63]. The phosphoinositide 3-kinase (PI3K) / protein kinase B (PKB) pathway is also involved in this regulatory mechanism due to its capacity to inhibit GSK-3β. Indeed, in *keap1* knockout mice, additional loss of the PI3K negative regulator *Pten* promotes an increase in Nrf2 levels through the inactivation of Gsk-3β [64]. In addition, protein kinase B (PKB) also phosphorylates Ser-40 in the Neh2 domain, dissociating NRF2 from KEAP1 and increasing NRF2 protein levels [65].

Another NRF2E3 ubiquitin ligase is the HMG-CoA reductase degradation protein 1 (HRD1), which interacts with NRF2 Neh4 and 5 domains and triggers NRF2 degradation under endoplasmic reticulum stress [66]. Recently, the cullin4/damaged DNA binding protein-1/WD Repeat Domain 23 (CUL4/DDB1/WDR23) was discovered as yet another E3 ligase of NRF2 that acts independently of CUL3/KEAP1 and competes for NRF2, suppressing NRF2 activity. WRD23 binds near the NRF2 DLG motif, but its role in NRF2 stability is still unclear [67] (Figure 3C).

Finally, IkB kinase β (IKKβ) is able to interact with KEAP1 promoting IKKβ degradation [68]. In basal conditions, KEAP1-Cul3-E3 ligase complex leads to IKKβ ubiquitination and degradation by the proteasome. In response to oxidative stress, however, IKKβ/KEAP1 binding is disrupted, promoting IKKβ stabilization and the phosphorylation of IkBα. Phosphorylated IkBα in turn binds and activates NF-kB. The consequence is the activation of a variety of NF-kB target genes involved in important processes, such as inflammation, tumor invasion and angiogenesis [69,70,71].

## 3. Functions of NRF2 in Lung Cancer. The Dual Role of NRF2

**Figure 4:**
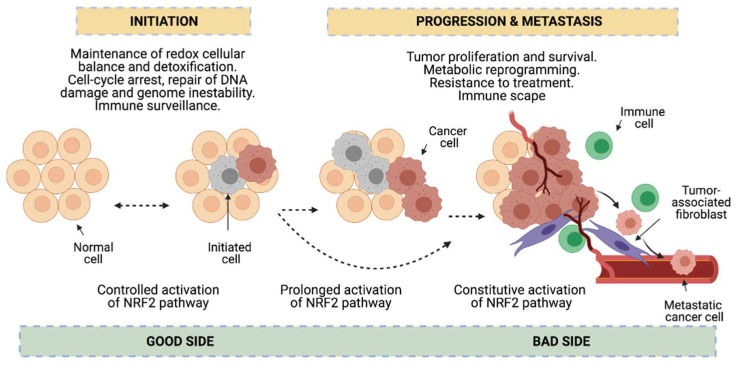
Dual role of NRF2 in cancer. NRF2 functions seem to be beneficial or prejudicial for tumorigenesis depending on the cancer stage. In early stages, NRF2 is protective in premalignant carcinogenesis and maintains redox cellular balance which assists in the detoxification process, DNA damage, cell-arrest, genome instability and immune surveillance (good side of NRF2 against cancer). However, prolonged or constitutive activation of NRF2 contributes to cancer progression and metastasis, since NRF2 favors tumor proliferation and survival, metabolic reprogramming, resistance to treatment and immune escape (bad side of NRF2).

Redox status imbalance commonly appears in cancer [72]. Tumor cells exhibit permanent high ROS levels due to the oncogene activation, increased metabolic rates, hypoxia, mitochondrial and/or peroxisomal dysfunction as well as anchorage-independent growth [51]. In this context, NRF2 plays a key role acting as a major regulator of the antioxidant response. Nonetheless, its functions can be beneficial or prejudicial for tumorigenesis depending on the cancer-stage in lung cancer cells. In early stages of tumorigenesis, NRF2 activity seems to be important for avoiding premalignant carcinogenesis, DNA damage and initial cancer mutations [25,73]. However, at advanced stages, some actions of NRF2 can promote carcinogenesis [25]. Activation of the NRF2 pathway could be advantageous to protect tumor cells from oxidative stress [73] (Figure 4). Lung cancer cells seem to acquire a high dependency on the NRF2 pathway for the maintenance of its malignant phenotype, a process called NRF2 addiction [74].

This dual role of NRF2 in cancer has been investigated in several animal studies. Tao et al. described in models of LUAD that the NRF2 pre-activation by sulforaphane prevents tumor initiation. Nonetheless, once the tumor is initiated, NRF2 activation promotes the growth of the pre-existing tumors, giving rise to larger tumors compared to non-treated tumors [75]. Satoh et al. found that after exposure to the carcinogen urethan, *Nrf2*-deficient mice have a relative increase in the number of tumor foci after 8 weeks of treatment, but by 16 weeks of treatment, these same animals show less advanced malignancy [76]. The same group also demonstrated that in *Keap1* knockdown mice, the growth of urethane-induced lung tumors is mitigated, thus preventing carcinogenesis. However, after transplantation, *Keap1*-knockdown mouse-derived cancer cells exhibited higher tumorigenicity compared to wild-type transplanted cells from control mice [77]. Thus, it is evident that NRF2 exhibits a dual role in cancer; however, there is still some ambiguity about its functions in this disease. In the following sections, the most important findings from NRF2’s beneficial (good side) or prejudicial (bad side) roles in NSCLC cancer will be summarized.

### 3.1. Good Side of NRF2 against NSCLC

NRF2 is able to protect cells from the oxidative stress generated during the transformation process by controlling several target genes which are mainly implicated in antioxidant defense and cell survival processes [13]. In this sense, NRF2 maintains an appropriate ratio of specific intracellular key antioxidants, such as reduced glutathione (GSH)/oxidized glutathione (GSSG), by controlling the expression of glutamate-cysteine ligase catalytic subunits (GCLC), glutamate cysteine ligase (GCL) or glutathione reductase 1 (GSR1) [78]. NRF2 also modulates the expression of several detoxification enzymes, such as glutathione peroxidase 2 (GPX2), thioredoxin 1 (TXN1), thioredoxin reductase 1 (TXNRD1), sulfiredoxin 1 (SRXN 1) and glutathione S-transferases (GSTs) [28]. All of them play important roles in the maintenance of cellular redox balance, which works against cancer progression.

In addition, NRF2 prevents DNA damage by regulating the expression of NAD (P) H Quinone Dehydrogenase 1 (NQO1) [79] required for the control of *breast cancer 1* (*BRCA1*) and *RAD51* recombinase (*RAD51*) mRNA levels; both proteins regulate homologous recombination (HR) during the repair of double-strand breaks (DSB) [80]. Accordingly, *Nrf2*-null mice are more susceptible to acute DNA damage [81] and in normal human lung fibroblasts, irradiation activates NRF2, which in turn reduces DSB levels [82]. These defense mechanisms against tumorigenesis managed by NRF2 have also been observed in additional animal studies [77,83,84]. For instance, Satoh et al. described that urethane-initiated LUAD have a smaller size when generated in *keap1*-knockdown mice compared to wild-type mice [77]. The protective function of NRF2 was corroborated in a Lewis lung carcinoma (3LL) mouse metastasis model, where the loss of *Nfe2l2* was linked to high metastasis capacity [85]. Recently, it was observed that cysteine dioxygenase type 1 (Cdo1) accumulation in LUAD generated in *Keap1^R554Q/R554Q^* mutant mice correlated with reduced tumor formation [86]. Finally, it was shown that some NRF2 activators, such as oltipraz, are able to block B(a)P-initiated LUAD in mice [87]. Similarly, several clinical studies done in patients with other cancer types (melanoma, prostate, colorectal and renal carcinomas) have demonstrated that the intervention by small molecules or phytochemicals, such as glucoraphanin and bardoxolone methyl, are able to activate NRF2 signaling, suppressing the risk of cancer progression [88,89].

NRF2 is also able to modulate the inflammatory response at the tumor site. NRF2 decreases the expression of pro-inflammatory cytokines such as tumor necrosis factor alpha (TNF-α), interleukin (IL)-1β (IL-1β), or IL-6 [51]. In the case of cellular immunity, NRF2 is also able to recruit natural killer (NK) cells that secrete the isoform D of IL-17, promoting tumor rejection [90]. Itoh and colleagues found that NRF2 up-regulation in the surrounding microenvironment or in hematopoietic cells suppresses lung tumor progression in *Kras*-driven LUAD tumors [91]. This finding prompted the idea that NRF2 activation in the tumor microenvironment could reduce cancer progression by enhancing the immune response against cancer [92]. In a *Keap1*-wt xenograft model of lung cancer, using the Nrf2 inducer bardoxolone triggered an increase in NRF2 expression and reduced the number of lung metastases [51]. Along the same line, Zhang et al. demonstrated that *Nrf2*-deficient mice show a higher number and volume of lung LUAD tumors compared to WT mice, a lower number of T cells (CD8 cytotoxic T cells and CD4 helper T cells) and increased amounts of some cytokines (CSF-1), chemokines (CCL9, CXCL12, CXCL1) and peptide antigens [59].

**Figure 5:**
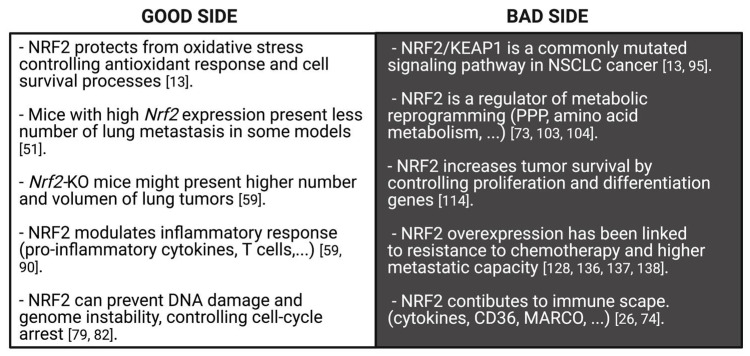
Good and bad sides of NRF2 against lung cancer. NRF2 exhibits a dual role in lung cancer. On one hand, NRF2 protects from oxidative stress generated in cancerous development by controlling genes implicated in antioxidant and cell survival processes, which maintain an appropriate level of intracellular antioxidants. Indeed, NRF2 prevents DNA damage, genome instability and cell cycle-arrest. Moreover, NRF2 modulates the inflammatory response through an increase of T cells. As a consequence, *Nrf2*-knockout mice show high number and volume of lung tumors and *Nrf2*-high mice present a small number of lung metastasis. On the other hand, the NRF2/KEAP1 pathway is a commonly mutated signaling pathway in human lung cancer and some pro-oncogenic functions of NRF2 provide an advantageous condition for the progression of cancer cells. NRF2 is an important regulator of metabolic reprogramming and cancer proliferation. Both processes are important for cancer progression. Concurrently, NRF2 overexpression has been correlated with lower sensitivity to chemotherapeutic drugs and with high metastatic capacity. Finally, NRF2 contributes to immune escape by inducing a decrease of pro-inflammatory cytokines.

Finally, some additional functions of NRF2 have been related to cancer prevention. NRF2 is involved in the control of cell cycle-arrest by regulating cyclin-dependent kinase inhibitors (CDKi) p15 (*Cdkn2a*) and p21 (*Cdkn1a*), in the prevention of genome instability [51], modulation of transcriptional initiation [49] and facilitation of aggresome formation during proteasomal stress [73]. Some in vitro studies show that NRF2 activation reduces LUAD cells’ capacity for migration [93,94]. Therefore, the implication from all the above-mentioned studies is that NRF2 may regulate a defense mechanism against tumor initiation, and also protect cells from cancer progression (Figure 5).

### 3.2. Bad Side of NRF2 in NSCLC Progression

As summarized in the above section, the NRF2/KEAP1 pathway has been considered as a tumor suppressor-signaling pathway due to its role in several defense mechanisms against tumor development. However, in the last decade, there has been growing evidence that this transcription factor has some pro-oncogenic properties. Activating mutations accumulate in some tumor types, suggesting that NRF2/KEAP1 may support an advantageous condition for cancer progression [13]. Although the first involvement of NRF2 in cancer was discovered in hepatocellular carcinoma [83], further experiments found elevated NRF2 protein levels in other malignancies, such as lung cancer [73,74]. *NFE2L2/KEAP1* is a commonly mutated signaling pathway in NSCLC sub-types [95] and GOF mutations of *NFE2L2* or LOF mutations of *KEAP1* have been often seen in this kind of malignancy [13]. Whereas *KEAP1* mutations were found in 17 % of LUAD and 12% of LUSC tumors, *NFE2L2* mutations are more frequent in LUSC (15 % of LUSC) than LUAD (3 % of LUAD) [10]. Globally, 26% of NSCLC tumors exhibit an increase in nuclear NRF2 expression [14]. Regarding other lung cancer sub-types, *Keap1* mutations have been found in an LCC tumor in mice [15], in SCLC (NCI-H1184, a human cell line) [16] and in some pulmonary large-cell neuroendocrine carcinoma (LCNECs) [17], although at a lower frequency than in LUSC or LUAD.

Interestingly, genetic analyses revealed that the *NFE2L2* mutational profile found in LUAD is significantly different from LUSC [96]. In fact, while *KEAP1* is mostly mutated in LUAD, *NFE2L2* is mainly affected in LUSC [97,98]; in the latter, *CUL3* is also significantly mutated [12,26]. Other genetic alterations in this signaling cascade also found in LUSC patients include single nucleotide polymorphisms (SNPs) in *KEAP1* and *CUL3* [99], an increased number of *NFE2L2* copies [100], *KEAP1* loss of heterozygosity (LOH) and *KEAP1* promoter methylations [101]. LUSC also shows additionally NRF2-complexed hypomorph (*ANCHOR*) mutations. While most mutations found in *KEAP1* show a reduced binding to NRF2, some, such as *KEAP1^R320Q^* and *KEAP1^R470C^,* exhibit an increased binding but fail to suppress NRF2 by forming a p62-dependent biomolecular complex. As a consequence, cells with these *KEAP1* mutations present moderately elevated levels of *NFE2L2*-dependent transcription [102].

This “bad side” action of NRF2 in NSCLC involves the regulation of enzymes related to metabolic reprogramming, where dividing cells conduct aerobic glycolysis (Warburg effect), an important process in cancer progression [74,103]. NRF2 can up-regulate pyruvate kinase (PK), a key enzyme in glycolysis [103]. Indeed, NRF2 is able to increase glucose uptake and redirect it to the pentose phosphate pathway (PPP), which is highly connected to the glycolysis route. This is made possible due to NRF2-mediated transcription of glucose-6 phosphate dehydrogenase (*G6PD*), 6-phosphogluconate dehydrogenase (*PGD*), transketolase (*TKT*) and trans-aldolase 1 (*TALDO1*) [73,104]. Furthermore, Best et al. found that *Keap1* mutant mice develop LUAD tumors with high levels of *Taldo1,* an enzyme that provides ribose-5-phosphate for nucleic acid synthesis and NADPH for lipid biosynthesis [105]. Simultaneous inactivation of *Keap1* and *Pten* genes in mice promotes the formation of LUAD and re-programming of metabolism to the PPP [106]. Correspondingly, reduced tumor growth has been reported in *Keap1* mutant NSCLC xenograft after *G6pd* and *Tkt* silencing [104].

NRF2 also regulates the expression of NADPH-producing enzymes (i.e., malic enzyme 1 (*ME1*), isocitrate dehydrogenase 1 (*IDH1*)) [73]. Surprisingly, NAPDH oxidase 2 (NOX2) and 4 (NOX4) are overexpressed in NSCLC [107,108,109] and generate superoxide and hydrogen peroxide, which in turn trigger NRF2 activation [109].

Another process clearly affected by NRF2 overexpression in cancer is amino acid metabolism, which facilitates the survival and proliferation of cancer cells under different stresses [73,74]. In this sense, studies using patient-derived xenografts (PDXs) and LUAD tumors from *Keap1*-deficient mice have shown an increased sensitivity to glutaminase inhibition (the enzyme that generates glutamate from glutamine), sensitizing KEAP1/NRF2 mutant NSCLC cells to radiotherapy [110,111]. Indeed, *liver kinase B1* (*LKB1*)-deficient cells combined with NRF2 activation promotes glutamine-addictive metabolism in *K-RAS* mutant LUAD [112]. Similarly, NRF2 activation in *KEAP1* mutant NSCLC lines promotes serine biosynthesis, required for the synthesis of key antioxidants, such as GSH. In addition, the NRF2 regulation of xCT, TXN and TXNRD1 promotes cysteine accumulation, a feature that correlates with poor prognosis in NSCLC cell lines and tumors [113]. This cysteine accumulation has been proposed as a metabolic liability in NSCLC cells, mainly in *KEAP1* mutant LUAD, by increased stabilization of cysteine dioxygenase 1 (CDO1) [86].

Altered lipid metabolism is another important NRF2-regulated metabolic feature in cancer. NRF2 reduces the expression of several fatty acid synthesis enzymes, such as fatty acid synthase (FASN), stearoyl CoA desaturase 1 (SCD1) and lipases, including in this last enzyme the phospholipase A2 Group VII (PLA2G7) [103].

The constitutive activation of NRF2 in cancer is also correlated with increased survival of tumor cells under unfavorable conditions because of the control by NRF2 of critical regulators of cell proliferation and differentiation [13,114]. NRF2 is able to control cell proliferation by increasing insulin-like growth factor-1 (*IGF1*), platelet derived growth factor C (*PDGFC*), and vascular endothelial growth factor C (*VEGFC*) levels [51]. Takahashi et al. found that the high activity of NRF2 in some NSCLC cell lines is key for efficient spheroid formation [115]. A new mechanism related to NRF2 contribution to increase proliferation was recently proposed: the activation of Notch Receptor 3 (NOTCH3). Surprisingly, this mechanism only was seen in *NFE2L2*-overexpressed NSCLC, raising the idea that the genes induced by NRF2 in cancer cells might be different from those induced in *NFE2L2*-WT background [116]. In addition, some authors found a correlation between NRF2 pathway alterations and poor survival. In fact, nuclear NRF2 expression has been associated with worse progress-free survival in NSCLC patients [14]. Lung tumors with high expression levels of *NQO1*, a *NFE2L2* target gene, have worse prognosis than those with wild-type *NQO1* [117]. Furthermore, overexpression of *CUL3* in LUAD patients (resulting in NRF2 degradation) has been related to a reduction of tumor growth in vivo and a better overall survival illustrating the involvement of NRF2 in LUAD progression [118]. Tong et al. also found that NRF2–negative and NQO1–negative NSCLC patient samples were correlated with better prognosis and disease-free survival [119].

Another family of NRF2 effectors are the aldo-keto reductases (AKRs, *AKR1B, AKR1C1/2* and *AKR1C3*) which are upregulated in many LUSC and some LUAD tumor biopsies with somatic mutations in the *NFE2L2* gene [120]. *AKR1C1* has been proposed as a target for the anti-tumor compound wentilactone A in SCLC cells [121]; indeed, the aldo-keto reductases are considered as biomarkers for NRF2 status in human tumors [120]. In this regard, *AKR1B10* gene overexpression has been also considered as a prognostic factor for poor recurrence-free survival in resected LUAD patients [122].

NRF2 also regulates proteins required for stemness, such as Aldehyde de-hydrogenases (ALDH), Sirtuin 1 (SIRT1) and others [123,124]. Li et al. described that EZH2 (enhancer of zeste homolog 2) inhibits lung cancer growth both in vitro and in vivo, and it does so by binding and repressing the *NFE2L2* promoter [125]. Additionally, tyrosine kinase receptors, such as insulin-like growth factor 1 receptor (IGF1R) and erb-B2 receptor tyrosine kinase 3 (ERBB3), were recently discovered to be also important for *KEAP1*-mutant lung cell growth [126]. Krall et al. showed that *KEAP1* loss is involved in the resistance of NSCLC cell lines with mutations in *EGFR, ALK*, *B-RAF* and *K/N-RAS* to selective inhibitors of these kinases [127].

The most frequent cause of mortality in lung cancer patients is metastasis. In this regard, several studies have related constitutive activation of NRF2 with this cancer stage [73]. Cells with *NFE2L2* overexpression have the ability to grow in an anchorage-independent manner, presenting a high metastatic capacity [128]. Aljohani et al. described that *NFE2L2^E63Q^* and *KEAP1^R601L^* mutations, both presenting constitutive activation of NRF2, are highly enriched in NSCLC metastasis [129]. This relation seems to be due to the regulation that NRF2 exerts on the expression of heme oxygenase 1 (encoded by *HMOX-1* gene). NRF2 can increase *HMOX-1* expression, and in turn that of the *BTB Domain and CNC Homolog 1* (*BACH1*) gene. By enhancing *BACH1* levels, NRF2 provokes the expression of pro-metastasis genes, such as those related to matrix metallopeptidases and CXCR4 in LUAD [130].

NRF2 can also promote lung cancer progression by regulating genes involved in angiogenesis [74,131], hypoxia [28,132], epithelial-mesenchymal transition (EMT) [74] and focal adhesion [133]. NRF2 is also able to regulate the cancer immune microenvironment. NRF2 can contribute to immune escape by controlling the expression of several cytokines [74], the scavenger receptors cluster of differentiation 36 (CD36) and macrophage receptors with a collagenous structure (MARCO) [26]. In agreement, *KEAP1* mutations in LUAD have been associated with reduced leukocyte infiltration [134] and *NFE2L2* mutant tumors exhibit low expression of different markers of immune response [135] (Figure 5).

### 3.3. Another Bad Side of NRF2 Activation: Resistance to Chemotherapy

NRF2 overexpression has been also linked to decreased sensitivity to chemotherapeutic drugs. *Nfe2l2* silencing in xenografts produces higher susceptibility to platinum-based chemotherapy compared to control xenografts [136]. Several reports have demonstrated that mutations in the NRF2/KEAP1 pathway are linked to worse outcomes after platinum-based chemotherapy [135,137,138]. The inhibition of tumor growth of A549 LUAD xenografts with paclitaxel was improved by inhibiting NRF2 pathway (through the administration of diosmetin) [139]. Frank et al. found that NSCLC patients with *KEAP1/NRF2* activating mutations do not respond to second/third line chemotherapy [97]; moreover, Ceston et al. found that LUSC patients with NRF2 active do not benefit from adjuvant chemotherapy comparing to the ones with the NRF2 unaltered [140]. All of these studies suggest that *KEAP1/NFE2L2* mutations might be used as a local recurrence risk predictor for chemotherapy.

The chemoresistance promoted by this transcription factor is due, at least in part, to NRF2 control of expression of drug transport genes, such as the *cysteine/glutamate antiporter system Xc-subunit* gene (*SLC7A11*) [28]. *SLC7A11* is overexpressed in different NSCLC cell lines and its silencing with shRNAs causes cell growth inhibition [141]. Similarly, another NRF2 target involved in chemoresistance is the multidrug resistance protein 3 (MDR3). In a previous study with the NSCLC cell lines A549 and H460 (both contain a *KEAP1* mutation), cells presented constitutively high levels of MDR3 [142]. Furthermore, in response to chemotherapy, NRF2 is able to inhibit apoptosis as it induces B-cell lymphoma 2 (Bcl-2) and Bcl-xL expression, which in turn reduces caspase 3/7 activation [143,144]. Furthermore, Chakrabarti et al. found that *glutamate-oxaloacetate transaminase 1* (*GOT1*) and *malic enzyme 1* (*ME1*) expression, both gene targets of NRF2, could be used as predictive markers in the treatment response to radiation therapy in NSCLC patients [145]. Finally, the homeodomain-interacting protein kinase 2 (HIPK2) has been recently reported as a new NRF2 target with anti-apoptotic functions [146].

The importance of NRF2 in drug resistance is also closely linked to the control that this transcription factor exerts on iron metabolism. In fact, NRF2 has a key role in the protection of lung cancer cells from ferroptosis, a cell death mechanism involving iron-dependent lipid peroxidation [51]. The involvement of NRF2 in this kind of cell death is caused by NRF2 control of the expression of iron pool-related genes, i.e., *ferritin heavy chain 1* (*FTH1*) [103], *glutathione peroxidase 4* (*GPX4*) and genes involved in glutathione and NADPH syntheses [147].

## 4. Mechanisms Conferring NRF2 Activation

**Figure 6:**
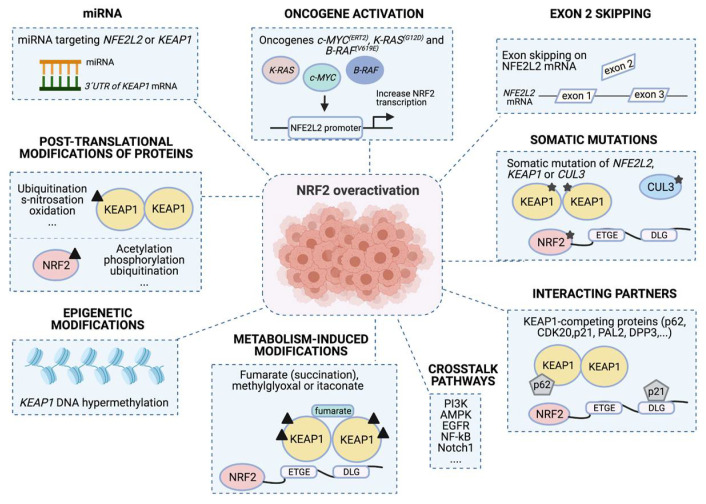
Molecular mechanisms confer NRF2 addiction. Different mechanisms can contribute to promote NRF2 over-activation. First, somatic mutations on NFE2L2/KEAP1/CUL3; second, differential slicing and loss of exon 2 during mRNA processing; third, mutations in other oncogenes, such as c-MYC (ERT2), K-RAS (G12D) and B-RAF (V619E) that increase NFE2L2 transcriptional levels. NRF2 can also be activated by: fourth, expression of different miRNAs that can regulate NRF2 and KEAP1 expression; fifth, post-translational modifications of NRF2 and KEAP1 that promote or suppress NRF2 activity; or sixth, epigenetic modifications, such as histone post-translation modifications in KEAP1 and NFE2L2 promoter regions that increase NRF2 accumulation. Finally, seventh: metabolites that can modify several residues in KEAP1, provoke NRF2 activation (metabolism-induced modifications); and eighth, NRF2 and KEAP1 interactions with other proteins (interacting partners) or pathways (crosstalk pathways) can also result in NRF2 activation.

The persistent activation of NRF2 in lung cancer cells is caused by different molecular changes, such as genomic alterations (genetic, epigenetic or oncogene signaling), transcription and translation abnormalities, post-translation modifications and/or altered interactions with other proteins [28,58,74]. All these molecular changes promote, in the end, the constitutive activation of NRF2 in lung cancer cells [58] and in turn NRF2 addiction (see Section 3) (Figure 6).

### 4.1. Somatic Mutations of NFE2L2/KEAP1/CUL3

The most common mechanisms promoting constitutive NRF2 activation are LOF mutations of *KEAP1* and GOF mutations of *NFE2L2* [74], which are frequently found in NSCLC sub-types [10,95] (see Section 3.2). In the case of *NFE2L2,* GOF mutations are mainly seen in the DLG and ETGE motifs [26,28]. In contrast, although *KEAP1* LOF mutations are mostly located in Kelch domain, they are also seen throughout the gene [58,134]. Some *KEAP1* mutations decrease NRF2 proteasomal degradation and hence cause NRF2 accumulation in the nucleus [6,26]. Other mutations, such as those causing *KEAP1* hypomorphic function (partial loss of gene function) [28] as well as “ANCHOR” or super-binder mutations, also induce constitutive NRF2 activation [44]. In fact, the *Keap1* deletion LUSC-like mouse model presents Nrf2 activation [21]. Concurrently, NSCLC cell lines with *KEAP1/NFE2L2* mutations present increased expression of some *NFE2L2* target genes, such as *HMOX1*, *GCLCM*, *TXN*, *GCLC*, *NQO1* or *GSR* [98]. In the case of *CUL3* mutations, they have been identified in the lung as well as hereditary type-2 papillary renal cell carcinoma [26,58].

For NRF2 activation, it is important to consider that other mutated genes that contribute to NSCLC development (*EGFR*, *TP53*, *KRAS*, *PTEN* and *PIK3CA*) [135,148] correlate with particular *NFE2L2/KEAP1* mutations. Mutations in *NFE2L2* usually co-occur with *PI3KCA* and *TP53* mutations [149,150]; meanwhile, *KEAP1* mutations co-occur more frequently with mutations in *K-RAS* or *STK11* [105,138]. *EGFR* mutations can coexist with *NFE2L2* mutations [148,151].

### 4.2. Exon 2 Skipping on NFE2L2 mRNA

Another important event that can occur during *NFE2L2* mRNA processing is the loss of its exon 2. The consequence of this differential splicing is the formation of *NFE2L2* aberrant transcripts that lack DLG or ETGE motifs, leading to a permanent nuclear location, and therefore promoting constitutive NRF2 activation [152]. This altered mRNA processing has been observed in NSCLC and head-neck cancer [153].

### 4.3. Oncogene Activation

Although the NRF2 pathway is mostly regulated at the protein level (by ubiquitination; see Section 1.2) [13], several molecular mechanisms and transcription factors exert control on its transcription levels. However, these processes have been less characterized [37].

Different mutations in several oncogenes, such as *c-MYC^(ERT2)^, K-RAS^(G12D)^* and *B-RAF^(V619E)^*, can affect *NFE2L2* transcriptional levels and/or activity [13,74]. Part of the pro-tumorigenic action of these oncogene mutations seems to be NRF2 activation [25,51,74]. Tao and DeNicola et al. identified enhanced *NFE2L2* mRNA levels with constitutive expression of *K-RAS^(G12D)^* [154,155] and showed that *K-RAS* binds to *NFE2L2* exon 1 and up-regulates its expression, conferring chemoresistance on NSCLC cells [154]. Moreover, around 30% of lung carcinoma cases with aggressive proliferation show *KEAP1* mutations, *K-RAS/H-RAS* mutations and *Tp53* LOF mutations [28]. Some studies suggest that *K-RAS^(G12D)^* and *B-RAF^(V619E)^* may increase *NFE2L2* transcription levels through Jun and Myc [155].

Other transcription factors that increase NRF2 transcription include Aryl hydrocarbon receptor/Aryl hydrocarbon receptor nuclear translocator (AHR/ARNT) [156] or NRF2 itself by its ARE element, the latter leading to a positive feedback mechanism [157].

### 4.4. miRNA

MicroRNAs (miRNAs) are short, single-stranded and non-coding RNAs that bind to the 3′-untranslated region of a gene transcript, promoting its mRNA degradation or inhibition of translation [13,74]. The first miRNA found to regulate *NFE2L2* expression levels was *miR-144*, whose expression decreases NRF2 protein levels [158]. Other miRNAs that suppress NRF2 activation in different cancers are *miR-507*, *miR-634*, *miR450a*, *miR129-5p, miR-340*, *miR-146b*, *miR-28*, *miR-153*, *miR142-5p*, *mi-R27a*, *miR-144*, *miR34a* and *miR-93* [28,58,156].

Other miRNAs have the ability to increase the expression of *NFE2L2* and/or its target genes. This is the case with *miR-155*, which increases the expression of some NRF2 target genes, such as *NQO1* and *HMOX1* genes, leading to resistance to arsenic trioxide (ATO) stress [159]. Increased expression of *NFE2L2* was observed by targeting the 3′UTR of *KEAP1* mRNA with *miR-24-3p*, *miR-7*, *miR-200a*, *miR-421*, *miR-141*, *miR-626* and *miR-873* [44,156].

Certain long non-coding RNAs are also involved in NRF2 activation: i.e., Urothelial Cancer Associated 1 (UCA1), HOX Transcript Antisense RNA (HOTAIR), Metastasis Associated Lung Adenocarcinoma Transcript 1 (MALAT1) or Taurine Up Regulated 1 (TUG1) [156].

### 4.5. Post-Translational Modifications

The post-translational modifications that can regulate NRF2 levels are acetylation, phosphorylation and ubiquitination [74]. In fact, NRF2 can be acetylated by CBP [160], acetyl transferase p300 [161] and hMOF (human males absent on the first), all of which result in increased NRF2 protein levels in the nucleus [162]. Correspondingly, NRF2 can be deacetylated by Sirtuin 1 (SIRT1) [160] or SIRT2, resulting in reduced NRF2 levels in the nucleus [74]. In addition, NRF2 can be stabilized by deubiquitination by the deubiquitinating enzyme 3 (DUB3) [163]. Recently, it was found that NRF2 could also be glycated, reducing its protein stability and binding to MAF [73].

KEAP1 can also undergo several post-translational modifications including ubiquitination, s-nitrosation, alkylation, glycosylation, oxidation, carbonylation, S-glutathionylation, succination and sulfhydrylation, which in turn affect NRF2 protein levels. In this sense, a recent report has described that deubiquitinating enzyme Ubiquitin Specific Peptidase 15 (USP15) inhibits the NRF2 activation through deubiquitination of KEAP1. In contrast, s-nitrosylation and oxidative KEAP1 modification induces NRF2 activation [44]. Indeed, it has been identified that KEAP1 can be glycosylated, although the consequences of this modification remain unclear [73]. Other modifications that can affect KEAP1 are malonylation, acetylation or palmitoylation, although their consequences are also still unknown [44].

### 4.6. Epigenetic Modifications

Histone post-translational modifications by EZH are another mechanism through which expression levels of *NFE2L2/KEAP1* can be regulated [125]. Epigenetic modifications have been described in *KEAP1* and *NFE2L2* promoter regions [28]. Whereas EZH inhibits *NFE2L2* expression and decreases NSCLC growth in vivo and in vitro [125], hypermethylation of *KEAP1* promoter inhibits its expression and results in increased NRF2 levels [164]. The latter modification has been related to poor clinical prognosis in NSCLC [165]. Indeed, Sparaneo et al., studying lung carcinoids (derived from the neuroendocrine system) (*n* = 47), found that whereas 50% exhibit *KEAP1* promoter methylation, 60% present *KEAP1* LOH; thus, in some tumors, both copies of *KEAP1* may be inactivated [101].

### 4.7. NRF2/KEAP1 Interacting Partners

Another way of affecting NRF2 function is though interactions with different proteins. Some proteins are able to bind to KEAP1 and disrupt its binding with NRF2, resulting in NRF2 activation. This is the case of p62/SQSTM1, which promotes KEAP1 degradation by autophagy and hence increases NRF2 activity [37]. Surprisingly, in this molecular axis, NRF2 is able to bind to the promoter region of p62, favoring KEAP1 degradation and in turn its own activation [166].

Other examples of interacting partners are those related to cell cycle regulators, such as cyclin dependent kinase 20 (CDK20). This cyclin has an ETGE motif which permits its binding to KEAP1 and results in an accumulation of NRF2 protein. In fact, overexpression of *CDK20* gene has been found in NSCLC. This overexpression was critical for the chemoresistance response in this malignancy [167]. Similarly, p21 is capable of interacting with NRF2 through its KRR motif. This binding disrupts NRF2 binding to KEAP1, promoting an activation of the NRF2 signaling pathway [168]. Similarly, other KEAP1 interacting partners bind to the ETGE motif and trigger NRF2 stabilization. These include the Wilms tumor gene on the X chromosome (WTX) or the partner and localizer of BRCA2 (PALB2) or DPP3 [28]. Ji et al. identified p53-induced protein with a death domain (PIDD) as a KEAP1-interacting partner, which promotes NRF2 stabilization and increases chemoresistance in H1299 NSCLC cells both in vitro and in vivo [169]. KEAP1 also interacts with Actin via its DGR domain, resulting in NRF2 nuclear translocation [44]. KEAP1 is also able to bind to Nestin via its Kelch domain-ESGE motif in NSCLC cells [170] and to the inhibitor of the apoptosis stimulating protein of p53 (iASPP) via the Kelch domain-DLT motif [171]. Other authors, such as Cheng et al., identified that the family with sequence similarity 129, member B or Niban-like protein 1 (FAM129B) competes for KEAP1 binding via both DLG and ETGE motifs, being that this process is linked to poor prognosis in breast (BRCA) and lung cancer (LUSC) [172]. Wang et al. showed that mitogen-activated protein kinase phosphatase 1 (MKP-1) binds to NRF2 Neh2 domain to inhibit its ubiquitination in NSCLC cells [173]. Recently, up to 46 new NRF2 interacting partners have been identified, which activate or repress NRF2 [174]. Further research is needed to understand their molecular mechanisms as well as consequences on NRF2/KEAP1 signaling pathways.

### 4.8. Metabolism-Induced Modifications

An example of a metabolism-induced modification is succination, which can affect KEAP1 function. In this reaction, fumarate modifies Cys residues in KEAP1 (possibly Cys151 and Cys288), impairing its ability to degrade NRF2 and leading to NRF2 activation [175]. Similarly, methylglyoxal (MGx), which is generated in glycolytic metabolism, induces the crosslink at Cys and Arg residues in KEAP1, triggering *NFE2L2* transcriptional program [176]. Finally, itaconate, a product of the tricarboxylic acid cycle (TCA) cycle, promotes alkylation of KEAP1 Cys residues (Cys151, Cys257, Cys288, Cys273 and Cyst 297) leading to NRF2 activation [73,177].

### 4.9. Crosstalk Pathways

Different pathways can modulate the activation or inhibition state of NRF2. Mitsuishi et al. demonstrated that activation of the PI3K/protein kinase B (AKT) pathway increases *NFE2L2* mRNA levels as well as its translocation to the nucleus [104]. The same effect has been described for AMP-activated protein kinase (AMPK), which is able to disrupt GSK-3β-NRF2 complex, allowing NRF2 nuclear translocation [178]. Similarly, EGFR induces the phosphorylation and ubiquitination of KEAP1, which in turn releases NRF2 [179]. Other pathways with the ability to promote NRF2 activation are the nuclear factor kappa light chain enhancer of activated B cells (NF-κB) [180] and NOTCH1, both increasing *NFE2L2* transcription. Additionally, *NOTCH1* gene contains, in its promoter, an ARE element, allowing for its transcriptional regulation by NRF2 [181,182].

Of particular interest is the puzzling effect of mutant *TP53* on NRF2 regulation. On one hand, when cells have high basal levels of *NFE2L2* target genes and present GOF *TP53* mutations, p53 is able to further increase *NFE2L2* transcriptional program. However, in cells with normal *NFE2L2* target levels, mutant *TP53* decreases the expression of *NFE2L2* target genes after oxidative stress [183]. In agreement, Tung et al. found *NFE2L2* increased expression levels in NSCLC cells with *TP53* mutations [150]. Another protein with the ability to inhibit the NRF2 pathway is the transcriptional repressor BACH1. BACH1 competes with NRF2 for ARE binding site of *HMOX1* gene, in turn decreasing *HO-1* levels [184].

## 5. Therapeutic Strategies for NRF2 Addiction

Classical NSCLC and SCLC treatment involves surgery (whenever possible), chemotherapy and radiotherapy. In recent years, targeted therapy (inhibitors of EGFR, ALK or ROS1 for NSCLC and inhibitors of PARP or DLL3 for SCLC) and immunotherapy (pembrolizumab, nivolumab, durvalumab, etc.) have been incorporated as therapeutic strategies for NSCLC and SCLC [185,186,187]. Many of these therapies have been applied to LUAD with optimal results; however, they are usually ineffective for LUSC. Since approximately 20% of lung cancers are LUSC and the survival rates for these patients remain unacceptably low, the development of targeted therapies for this tumor sub-type is critical [12]. Towards this end, NRF2 could prove to be a promising candidate for LUSC targeted therapy.

The emergence of drug resistance is frequent and it unmasks the need to find new compounds capable of avoiding these side effects [188]. In this regard, numerous studies are focused on the mechanism of resistance to each particular treatment. Recent studies point at the NRF2/KEAP1 pathway as a molecular mechanism linked to the emergence of resistance to chemotherapy treatment resistance, making this transcription factor an ideal target to recover drug sensitivity [97,135,137,138,140]. For this reason, the development of new therapeutic inhibitory strategies for NRF2-addicted lung cancer cells has attracted significant interest. Among them, the use of NRF2 inhibitors, immunotherapies for patients with active NRF2-bearing tumors or inhibitors of downstream NRF2 effectors are being widely studied [74].

### 5.1. Direct NRF2 Inhibitors

Several small molecules have been shown to inhibit NRF2 activity in tumors harboring *KEAP1* or *NFE2L2* mutations [26]. Although some of these direct NRF2 inhibitors have been widely used in pre-clinical studies, many of them are of poor efficacy in the clinic, showing low specificity and bioactivity [74]. Moreover, the molecular mechanism of some of these is still not well understood [37].

One of these direct NRF2 inhibitors is brusatol, which was identified as an NRF2 inhibitor in 2011 [189]. This compound is able to increase the response to irradiation combined with chemotherapeutic drugs (such as cisplatin) [190], thus reducing cancer cell proliferation in A549 LUAD cells in vitro and in vivo. Although brusatol enhances the poly-ubiquitination of NRF2, the molecular mechanism of its action is still unknown [189]. Later studies showed that brusatol has low specificity for NRF2 as this compound decreases the expression level of many other short half-life proteins, acting as a general inhibitor of the translation machinery [191]. Another inhibitor used in NSCLC, both for in vitro and in vivo studies, is the flavonoid luteolin [192,193]. Luteolin accelerates *NFE2L2* mRNA turnover and sensitizes cells, such as A549, to chemotherapeutic drugs (oxaliplatin, bleomycin and doxorubicin) [192]. Moreover, when combined with cisplatin, it is more effective in reducing tumor growth in A549 LUAD xenografts than cisplatin alone [193]. This compound has been also used in combination with ascorbic acid for the treatment of patients with *NFE2L2* mutations [194]. Other studies have shown that luteolin, in low doses, can activate *NFE2L2* and *HO-1* genes in HepG2 liver hepatocellular cells [195]. Further studies are needed to determine the specific mechanism of luteolin action [74].

Several nuclear factors, such as RXRα [38], retinoic acid receptor alpha (RARα) [196], estrogen receptor alpha (ERα) [197], peroxisome proliferator-activated receptor-γ (PPARγ) [198] or GR [35] have been shown to inhibit the NRF2 pathway. RARα, in the presence of the ligand all-trans retinoic acid (ATRA), forms a complex with the Neh7 domain of NRF2, blocking its ARE binding [38,196]. Similarly, bexarotene is another agonist described for RXRα [199]. Clobetasol propionate (CB) is also an interesting glucocorticoid candidate that was identified from a clinical compound library. CB interferes with NRF2 nuclear translocation via GSK-3β. CB promotes the poly-ubiquitination of NRF2 by β-TrCP, which in turn suppresses the growth of *KEAP1* mutant NSCLC lung cancer xenografts [200].

Other groups have carried out alternative chemical screening approaches to find small molecule inhibitors for NRF2 [26]. ML385, ARE expression modulator 1 (AEM1) and 4-(2-Cyclohexylethoxy) aniline (IM3829) were identified as synthetic NRF2 inhibitors in NSCLC lines [201,202,203]. ML385 binds to the Neh1 domain of NRF2 and blocks the ARE binding of NRF2, increasing the efficacy of some chemotherapeutic drugs (carboplatin, doxorubicin and paclitaxel). However, its selectivity for NRF2 has not yet been evaluated [201]. In the case of AEM1, it reduces NRF2 activity without altering NRF2 or KEAP1 protein levels and its combination with doxorubicin sensitizes A549 LUAD cells to etoposide and 5-fluorouracil [202]. Finally, IM3829 reduces *NFE2L2* mRNA levels and in combination with radiation, it inhibits the growth of NSCLC xenografts [203].

Some other NRF2 pathway inhibitors used in in vitro studies are trigonelline [204], chrysin (5,7-digydroxyflavone) [205], apigenin (4′,5,7-trihydroxyflavone) [206], halofuginone [207], cordycepin [208] or PHA-767491 and AZ-628 [209].

More recently, newly identified NRF2 inhibitors have been tested in NSCLC lung cells. This is the case for RNA-binding motif protein 47 (RBM47) [210], 3′,4′,5′,5,7-pentamethoxyflavone (PMF) [211], 4-methoxychalcone (4-MC) [212], triptolide [213], homoharringtonine [214], convallatoxin [215], diosmetin [139], flumethasone [216], coroglaucigenin (CGN) [217] and kaempferol [185]. Other studies have focused on disrupting NRF2/KEAP1 binding. This is the case for some potent phytochemicals, such as 3-(Dimethylamino)-3-imino-*N*-(4-methylphenyl) propanamide or phlorizin [218] or K67 (*N*-[2-acetonyl-4-(4-ethoxybenzenesulfonyla-mino) naphthalene-1-yl]-4-ethoxybenzenesulfonamide), that disrupt KEAP1-p62 binding [219]. Although most of them are effective in reducing NRF2 expression, more studies are needed to clarify their specificity and action in in vivo models. Moreover, it is still early to know the real possibilities of these inhibitors for cancer treatment due to the lack of clinical studies.

Finally, computational methods are being used to identify new chemical compounds capable of activating (2-nitrofluorene, resorcinol, etc.) or repressing (dexamethasone, sulfisoxazole, etc.) the NRF2 expression in human cells [220]. Further specificity and efficacy tests in lung cancer clinical trials are needed to validate the beneficial effects of NRF2 inhibition, either by itself or on sensitizing lung cancer to chemotherapy [134].

### 5.2. Immunotherapy for Active-NRF2 Tumors

Tumors escape surveillance and detection expressing PD-L1, which interacts with T cells and suppress the antitumor T cell response [221]. PD-L1 is directly controlled by NRF2 [222] and the tumor microenvironment of NRF2-active lung cancer cells present high levels of immunosuppressive proteins such as PD-L1 [106]. Interestingly, increased PD-L1 expression is associated with *NFE2L2* mutations in LUSC and *KEAP1* mutations in LUAD. These findings suggest that *NFE2L2/KEAP1* mutations/activation could be of benefit for LUSC and LUAD treatment by immunotherapy [223,224]. Furthermore, CTLA-4, which acts as a negative regulator of T cell response, has been a topic of research in NSCLC [221]. Indeed, sensitivity to immunotherapy was detected in NRF2-active *Keap1* and *Pten* LUAD-like mice models that present high levels of PD-L1. When LUAD in these mice were treated with cycles of anti-PD-L1/anti-Ctla-4 compounds, they achieved a surprising reduction in tumor burden. Further research is needed to verify the correlation between immunotherapy and NRF2 pathway activation in clinical trials, although this study suggests that high NRF2 activity tumors, particularly LUAD, could benefit from this treatment [106]. Therefore, activation of NRF2 could help to increase efficacy of immunomodulatory compounds [74].

### 5.3. Inhibitors of NRF2 Downstream Effectors

Inhibition of NRF2 downstream effectors combined with conventional therapy may result in an increase in patients’ progression-free survival rate. For instance, serine biosynthesis inhibition (CBR-5884) [225]; glutaminolysis inhibition (CB-839) [226]; PPP inhibition (polydatin) [227]; IL11 inhibition; or glutathione synthesis inhibition using, e.g., BSO (buthionine sulfoximine), 2-AAPA, sulfasalazine, or erastin [228,229,230,231,232].

*In vivo* studies in *K-RAS*-driven human LUAD xenografts and PDXs exhibiting *KEAP1* mutations show a reduction in tumor growth after treatment with glutaminase inhibitor CB-839 [110]. Most likely, targeting some metabolic pathways downstream of NRF2 could be a good strategy for NRF2-active lung cancer cells [134]. In a library screen, Mattheus et al. identified new possible inhibitors for NRF2-targets (lyngbyabellin A, grassypeptolide A and dolastatin 12) that reduce *NFE2L2*-target gene expression in MDA-MB-231 breast cell line and A549 cell line [233]. At the same time, for pathways that crosstalk with NRF2, pre-existing compounds for these pathways can be used in combination with NRF2–directed compounds [13]. In fact, a phase I trial combining MLN0128 (sapanisertib), an mTOR inhibitor, and CB-839, a glutaminolysis inhibitor, is ongoing in patients with advanced NSCLC (KRAS-mutant LUAD and LUSC) having *NFE2L2/KEAP1* mutations [234].

## 6. Conclusions

Overall, it is noteworthy that while NRF2 exerts cytoprotective effects in the prevention of malignant transformation in healthy tissues, once some tumor types are generated, NRF2 is also important in maintaining the cancerous state by protecting cancer cells from environmental ROS and limiting the damage induced by chemotherapy. This dual role of NRF2 discussed here in the context of NSCLC appears to be dependent on the stage of the tumor. NRF2 functions as a tumor suppressor generally at tumor initiation stages, while NRF2 pro-oncogenic functions are usually found at advanced stages of the tumor. Several reports have determined that lung tumor cells acquire an NRF2 overexpression dependency for maintenance of its malignant phenotype, a process labeled NRF2 addiction. The tumor protection exerted by NRF2 supports the cancer growth via multiple molecular mechanisms. Further research on this dual role of NRF2 is needed to clarify its functions in each cancer stage. The use of human samples, such as human biopsies or 3D cultures (organoids), could be helpful in this effort.

Therefore, it is reasonable to assume that blocking NRF2 activity in fully malignant lung tumors with constitutively active NRF2 may be an important strategy for the treatment of this disease. While previous studies have tried to provide direct NRF2 inhibitors that regulate its levels or action, currently, none have yielded strong and efficient results. Moreover, immunotherapy directed by NRF2 activation status has to be considered, since NRF2-addicted lung cancer cells exhibit high levels of immunosuppressive proteins, such as PD-L1. Nevertheless, it is still early for its use in patients due to the absence of clinical studies. Other therapeutic strategies not focused on direct NRF2 inhibition may be considered; i.e., the exploration of indirect methods of inhibition, such as upstream and/or downstream protein kinases, can be an open new therapeutic field for lung cancer patients. In this regard, screening methods involving gene-editing technologies, such as the CRISPR-CAS system, can be of great help to define new NRF2 regulators. Furthermore, the blockade of other genetic regulators, such as microRNAs, long non-coding RNAs or interference with interacting partners could also be considered as viable targets for NRF2-addicted lung cancer cells.

## Data Availability

Not applicable.
